# Supplementation with Bioactive Compounds Improves Health and Rejuvenates Biological Age in Postmenopausal Women

**DOI:** 10.3390/biom15050739

**Published:** 2025-05-20

**Authors:** Estefanía Díaz-Del Cerro, Judith Félix, Mª Carmen Martínez-Poyato, Mónica De la Fuente

**Affiliations:** 1 Department of Genetics, Physiology and Microbiology (Unity of Animal Physiology), Faculty of Biological Science, Complutense University of Madrid (UCM), 28040 Madrid, Spain; jufelix@ucm.es (J.F.); mariacmpoyato@gmail.com (M.C.M.-P.); 2 Institute of Investigation 12 de Octubre (imas12), 28041 Madrid, Spain

**Keywords:** postmenopausal women, dietary supplement, immune functions, oxidative state, stress hormones, biological age

## Abstract

Aging involves immune system deterioration (immunosenescence) and increased oxidative stress, both associated with morbidity and mortality. Menopause accelerates aging, highlighting the need for strategies to mitigate its effects in postmenopausal women. This study assessed the impact of daily oral supplementation for one month with 39 bioactive compounds (UNAMINA)—including amino acids, vitamins, and antioxidants—on immune function, redox parameters, stress-related hormones, and biological age in healthy postmenopausal women. Peripheral blood samples were collected before and after supplementation to analyze lymphocyte and neutrophil functions (adherence, chemotaxis, natural killer cell antitumor capacity, and lymphoproliferative response to mitogens), oxidative stress markers (antioxidant defenses such as glutathione peroxidase (GPx) and reductase activities, reduced glutathione (GSH) concentrations, as well as oxidants such as oxidized glutathione (GSSG), and lipid peroxidative damage) in blood cells, and stress-related hormones (dehydroepiandrosterone (DHEA) and cortisol) in plasma. Supplementation improved all immune cell functions and decreased oxidative stress (increasing antioxidants defenses such as GPx activity and GSH concentration and decreasing GSSG amount) and cortisol concentrations, whereas those of DHEA increased. The biological age also decreased. The results suggest that these bioactive compounds may be a beneficial strategy for promoting healthier aging in postmenopausal women by enhancing immune function, reducing biological age, improving redox balance, and regulating stress hormones.

## 1. Introduction

The aging process, which starts at adult age, is characterized by a decline in organism’s functions, especially those of homeostatic systems such as the immune system. Since dynamic balance, so-called homeostasis, is necessary for the maintenance of health, its age-related deterioration increases the risks of morbidity and mortality. The immune system, which is known as the best protector against pathogens and malignant cells, is also crucial in maintaining health and in modulating how a subject ages [1,2,3]. However, during the aging process, this system undergoes changes, which include both decreases and increases in some of its functions, with this restructuring known as “immunosenescence”. Moreover, several immune functions have been proposed as markers of individual aging rates or biological age, leading to the development of the Immunity Clock to determine biological age [3,4].

There are many aging theories, but one of the most accepted today is Harman’s theory of oxidation [5]. According to this theory, the aging process is a consequence of the accumulation of oxidative compounds produced by our cells because of the necessary use of oxygen to carry out their functions. To prevent oxidative damage, cells have developed a wide variety of antioxidant mechanisms, making it crucial to achieve a balance between oxidative compounds and antioxidant defenses to maintain proper body functionality and health. However, this balance is broken during the aging process, characterized by an increase in oxidant compounds and a decrease in antioxidant defenses. This oxidative stress leads to cell damage and worsening of different functions of the body, with a loss of homeostasis [2]. In fact, the oxidative stress situation of total blood cells in humans is related to their rate of aging [6].

It has been demonstrated that the maintenance of health, and consequently the rate of aging, depends mainly on lifestyle factors [2,3]. In this context, many studies have tried to find lifestyle strategies that slow down the aging process as well as delay its consequences. Among these strategies, we find nutrition [7,8,9,10]. In this context, dietary interventions have been the most researched non-pharmacological treatment that appears to counteract aging-related diseases, such as obesity, atherosclerosis, diabetes, and chronic disorders [11,12,13]. One example of this type of intervention is dietary supplements [14].

Dietary supplements include vitamins, minerals, essential fatty acids, amino acids, flavonoids, and herbs. All of them are safe substances to maintain health, but the concentrations used are also important [14]. Several studies have shown that the ingestion of diets with adequate amountsof antioxidants such as vitamins E and C, ß-carotene, polyphenols, and others can retard or prevent oxidative damage [7,15] and, therefore, the general physiological impairments associated with aging, particularly immunosenescence [16]. This suggests that these diets are a good way to improve the immune system in elderly subjects [2,7,17], which is very important because it is known that older people show the highest risk of both poor nutrition (with insufficient intake of these micronutrients) and increased oxidative stress [2,6,7,18,19].

Moreover, aging is associated with alterations in neuroimmunoendocrine responses to stress, in particular in the function of the hypothalamus–pituitary–adrenal (HPA) axis, which is crucial for regulating anxiety and stress responses. In this way, an inadequate stress response is one of the conditions that accelerates the aging process, accompanied by an alteration in homeostatic systems and their communication [20,21]. It has been suggested that one of the contributing factors to this exacerbated stress effect on aged organisms is their inability to end glucocorticoid production in response to stress [22]. This compromises the adequate immune function [23,24,25,26], increases the amounts of oxidant compounds [27,28] and, consequently, accelerates aging [28,29]. In this context, several studies have shown that dietary supplements at appropriate doses can act on the HPA axis and decrease exacerbated cortisol production [30,31,32].

In addition, menopause is an endocrine transition in which hormonal changes impact the brain, increasing the risk of mood disorders [33], with high levels of anxiety and perceived stress [34,35]. Moreover, menopausal women as well as ovariectomized rats and mice (a good model for mimicking human menopause) show immunosenescence and high oxidative stress situations [36,37,38,39]. Due to all of these alterations, it is accepted that menopause leads to the acceleration of aging [40,41].

Given the above and that in a previous preliminary study we demonstrated the positive effect of taking a supplement with 39 bioactive compounds (UNAMINA), including amino acids, vitamins, and antioxidants, for 1 month in some immune functions and biological age [4], in the present study we have investigated the effect of taking a similar supplement on a greater number of immune functions, as well as in several oxidative stress parameters of peripheral blood cells, and the amount of plasmatic stress hormones in healthy postmenopausal women. In addition, we have determined the biological age using the Immunity Clock [4].

## 2. Materials and Methods

### 2.1. Experimental Groups and Experimental Design

The participants were 15 postmenopausal women aged 59.3 ± 6.5 with similar demographic characteristics and lifestyle (Table 1), as well as a similar clinical history (Table 2).

Participants took the dietary supplements UNAMINA A, B, and C, containing 39 bioactive compounds, for 1 month (Table 3). The UNAMINA product is sold exclusively online and in medical consultations, and it was designed by Dr. Mª Carmen Martínez-Poyato within the framework of her company, Inside Beauty 07 S.L. (Murcia, Spain). UNAMINA A is a combination of 8 amino acid precursors of collagen with high concentrations of glycine (glycine, L-lysine, L-arginine, L-proline, L-leucine, L-isoleucine, L-valine, L-tryptophan) and hydrolyzed collagen, as well as methyl-sulfonyl-methane, magnesium citrate, calcium carbonate, and sucralose. Supplement UNAMINA B is a combination of amino acids, vitamins, antioxidants, micronutrients, and other compounds with relevant roles in cellular metabolism (L-ornithine, L-beta-alanine, N-acetyl L-cysteine, L-threonine, L-histidine, L-glutamine, L-taurine, thiamine (B1), riboflavin (B2), niacin (B3), pantothenic acid (B5), pyridoxine (B6), Biotin (B8), folate (B9), cobalamin (B12), zinc sulphate hydrate, sodium selenate, magnesium, manganese sulphate, chondroitin sulphate, glucosamine sulphate, sodium hyaluronate, and ferrous fumarate). Supplement UNAMINA C is constituted of cell metabolism stimulants (citric, malic, and ascorbic acids). The participants took one sachet of UNAMINA A per day, dissolved in water; one capsule of UNAMINA B taken in the morning; and two sachets of UNAMINA C daily, one in the morning and one in the evening, both dissolved in water, for 1 month.

Before and after taking the oral supplement for 1 month, 12 mL of blood was drawn between 9:00 and 10:00 a.m. to avoid variations due to circadian rhythm. The inclusion criterion was that they were postmenopausal women in healthy condition, which was defined as the absence of pathology or findings of clinical significance in general laboratory parameters. The exclusion criteria were severe general pathology (autoimmune diseases, cancer, anemia, severe allergies, dementia or cognitive alteration, chronic respiratory disease, hypertension, and diabetes); consumption of excess alcohol or drugs, vitamins, antioxidants, or any drug influencing the immune system; as well as poor collaboration. Women with mild or moderate conditions that did not require pharmacological treatment affecting the immune system or oxidative balance were not excluded. The Human Ethics Committee of the 12 de Octubre Hospital (Madrid, Spain) approved this study. In addition, the study was explained to the participants, and their consent was obtained in writing.

### 2.2. Survey on the Quality of Sleep, Diet, and Physical Activity

A survey was conducted to assess the sleep quality, diet, and physical activity among participants. The survey consisted of three sections, each rated on a 1 to 10 scale, where 1 indicated strong disagreement and 10 indicated strong agreement. The surveys were completed at initial assessment (7 days before UNAMINA supplementation) and 1 month after commencement of UNAMINA intake.

The Sleep Quality Survey categorized scores into three groups: deficient (1–4) for those sleeping less than 5 h per night, experiencing frequent awakenings, feeling tired upon waking, and having difficulty falling or staying asleep; acceptable (5–7) for those sleeping 5–7 h per night, with occasional awakenings and mild morning fatigue that does not significantly affect daily activities; and optimal (8–10) for those sleeping 7–9 h per night, experiencing deep and restorative sleep with few interruptions and feeling well-rested upon waking.

The Diet Survey categorized scores into three groups: deficient (1–4) for those with a high intake of processed foods, sugars, and unhealthy fats, low consumption of fruits, vegetables, and proteins, and irregular meal patterns; acceptable (5–7) for those with moderate consumption of fruits and vegetables and occasional intake of processed or unhealthy foods; and optimal (8–10) for those following a balanced diet with a high intake of fruits, vegetables, and quality proteins, low consumption of processed foods and sugars, proper hydration, and regular meal schedules.

The Physical Activity Survey also classified scores into three groups: deficient (1–4) for those leading a sedentary lifestyle, engaging in little or no weekly physical activity, experiencing frequent fatigue, and spending long hours sitting with few active breaks; acceptable (5–7) for those engaging in occasional physical activity (2–3 times per week) and alternating between active and sedentary periods; and optimal (8–10) for those exercising at least 4–5 times per week, including strength and cardiovascular activities.

### 2.3. Perceived Scale Stress (PSS)

Psychological stress was assessed using the Perceived Stress Scale (PSS-10), a 10-item questionnaire designed to evaluate an individual’s perception of stress over the past month. Responses were recorded on a Likert-type scale ranging from 0 to 4 (0 = never, 1 = rarely, 2 = sometimes, 3 = fairly often, 4 = very often), with total scores spanning from 0 to 40. Higher scores indicated greater perceived stress. Stress levels were categorized as low (0–13), moderate (14–26), and high (27–40). The PSS-10 has demonstrated strong reliability and internal consistency, with reported values between 0.84 and 0.86 [42]. Participants completed the questionnaire at baseline (7 days before starting UNAMINA supplementation) and again one month after beginning UNAMINA intake.

### 2.4. Hamilton Anxiety Rating Scale (HAM-A)

The HAM-A is a widely used, well-validated tool consisting of 14 items designed to assess the severity of a patient’s anxiety [43]. Each of the 14 items in this clinician-rated measure is rated on a 5-point scale, ranging from 0 = not present to 4 = severe, with total scores categorized as follows: 0–17 = mild anxiety, 18–24 = mild to moderate anxiety, 25–30 = moderate to severe anxiety, and 31–56 = very severe anxiety. The HAM-A was completed at the initial assessment (7 days before UNAMINA supplementation) and 1 month after the commencement of UNAMINA intake.

### 2.5. Collection of Human Blood Samples and Isolation of Lymphocytes and Neutrophils

Peripheral blood samples (12 mL) were collected by venous puncture in sodium citrate-buffered Vacutainer tubes (BD Diagnostic, Madrid, Spain). Plasma and whole blood cells were separated by centrifugation to assess cortisol and DHEA concentrations in plasma and oxidative parameters in whole blood cells. Neutrophils and mononuclear cells (principally lymphocytes) were isolated from complete blood by density gradient centrifugation, following a previously described method [3].

### 2.6. Immune Functions

The following immune functions were assessed both before and after taking the oral supplement.

#### 2.6.1. Adherence

To assess the adhesion capacity of neutrophils and lymphocytes, a modified version of the previously described method [44] was used. This method simulated in vitro cellular adhesion to the endothelium. Briefly, whole blood was diluted 1:1 with Hank’s solution and placed in an adhesion column, consisting of a Pasteur pipette filled with 50 mg of nylon fibers at a height of 1.25 cm. After 10 min, the effluent that was drained by gravity was collected and mixed with Türk’s solution (1:9 ratio), which differentiated cell morphology and lysed erythrocytes. Neutrophils and lymphocytes were counted using optical microscopy at 40× magnification before and after passing through the adhesion column. The difference between the initial count and the count after passing through the column represented the number of cells that adhered, which was used to calculate the adhesion index.

#### 2.6.2. Chemotaxis of Neutrophils and Lymphocytes

Chemotaxis capacity was evaluated using a modified version of the Boyden chamber method, following previously established protocols [3,45]. This technique assesses the ability of immune cells to migrate toward an infectious stimulus, simulated by a chemotactic agent. The Boyden chamber consists of two compartments separated by a nitrocellulose filter with 3 μm pores. The upper compartment contained 300 μL of leukocyte suspension, while the lower compartment held 400 μL of the chemotactic agent, the formylated peptide (fMet-Leu-Phe) from E. coli, at a concentration of 10^−8^ M. After 3 h of incubation at 37 °C and 5% CO_2_, cells attached to the filter were fixed (methanol 50% and ethanol 75%) and stained (azur-eosin-methylene blue solution, GIEMSA, Panreac Applichem, Madrid, Spain). The Chemotaxis Index (C.I.) for each cell type was determined by counting the number of cells that migrated to one-third of the lower surface of the filter using optical microscopy (×100).

#### 2.6.3. Phagocytic Neutrophil Capacity

The phagocytic capacity of neutrophils was evaluated using a previously established method [3], which measures the ability of phagocytic cells to ingest inert particles, such as latex beads, as an indicator of their in vivo function. Aliquots of 200 μL of neutrophil suspension were incubated on migration inhibition factor plates (Sterilin, Teddington, UK) for 30 min, the adherent monolayer was washed with phosphate-buffered saline (PBS) at 37 °C, and 20 μL of latex beads (1.09 μm diluted to 1% PBS, Sigma-Aldrich, Madrid, Spain) was added. After 30 min of incubation, neutrophils were fixed (methanol 50%) and stained with azur-eosinmethylene blue solution (GIEMSA, Panreac Applichem, Madrid, Spain). Phagocytic activity was assessed using two parameters: the Phagocytic Index, defined as the number of latex beads ingested by 100 neutrophils, and Phagocytic Efficiency, representing the percentage of neutrophils that ingested at least one latex particle. Both were determined using optical microscopy (×100).

#### 2.6.4. Natural Killer (NK) Cell Cytotoxic Activity

Natural killer (NK) cell cytotoxicity was assessed using a colorimetric enzymatic assay (Cytotox 96™ Promega, Boehringer Ingelheim, Ingelheim, Germany) that measures lactate dehydrogenase (LDH) release resulting from the lysis of target cells (K562 human lymphoma cell line) using tetrazolium salts [3]. Effector cells (lymphocytes) were added at a concentration of 10^5^ cells per well in U-bottomed 96-well culture plates, while target cells were seeded at 10^4^ cells per well, maintaining an effector-to-target ratio of 10:1. Following a 4 h incubation, LDH activity was determined by adding the enzyme-substrate and measuring the absorbance at 490 nm. The extent of tumor cell lysis (% lysis) was calculated using the following equation:

$$\%\;of\;lysis=\frac{E-ES-TS}{TM-TS}\times 100$$

where E represents the mean absorbance in the presence of both effector and target cells, ES corresponds to the mean absorbance of effector cells alone, TS is the mean absorbance of target cells alone, and TM denotes the maximum absorbance obtained after incubating target cells with lysis solution.

#### 2.6.5. Lymphoproliferation

Lymphocyte proliferative capacity was evaluated using a method previously described [3]. The assay was conducted under both basal and stimulated conditions using the mitogens phytohemagglutinin (PHA) and lipopolysaccharide (LPS) (1 μg/mL each, Sigma-Aldrich, Madrid, Spain). Mononuclear leukocyte suspensions were adjusted to 10^6^ lymphocytes/mL in RPMI medium (Gibco, Burlington, ON, Canada) supplemented with gentamicin (1 mg/mL, Gibco, Burlington, ON, Canada) and 10% heat-inactivated fetal bovine serum (Gibco, Burlington, ON, Canada); (inactivated at 56 °C for 30 min). Aliquots of 200 μL were dispensed into 96-well plates (Costar, Cambridge, MA, USA), and 20 μL of PHA or LPS were added to the respective wells, while control wells received 20 μL of supplemented RPMI medium. After 48 h of incubation, 0.5 μCi/well of ^3^H-thymidine (Dupont, Boston, MA, USA) was added, followed by an additional 24 h incubation. The cells were then harvested using a semi-automatic harvester, and thymidine incorporation was measured using a beta counter (LKB, Uppsala, Sweden) for 1 min. The results were expressed as ^3^H-thymidine uptake (counts per minute [c.p.m.]) for both basal and stimulated conditions. Lymphoproliferation capacity (%) was calculated by setting the c.p.m. of basal conditions as 100% and determining the relative increase in response to stimulation.

### 2.7. Oxidative Stress Parameters

The blood was centrifuged at 1300× *g* for 20 min to separate blood cells from plasma. The sediment of total blood cells was reconstituted with RPMI+ medium and frozen at −80 °C until its use for the assessment of the following oxidative stress parameters.

#### 2.7.1. Glutathione Reductase Enzyme Activity

Glutathione reductase (GR) activity was measured using spectrophotometry based on the method described by Massey and Williams (1965) [46], with slight modifications [47]. This method is based on the ability of GR to reduce oxidized glutathione. Briefly, the cells were sonicated in 50 mM phosphate buffer with EDTA at pH 7.4 (previously helium-bubbled), and the samples were then centrifuged. The supernatants were collected, and the oxidation of NADPH was monitored, which was observed as a decrease in absorbance over time at 340 nm. The GR activity, expressed in mU/mg protein, was calculated using the following equation:

$$\;GR\;activity\;(\frac{mU}{mg\;protein})=\frac{GR\;activity\times V_{t}\times F}{E\times V_{m}\times X}$$

where V_t_ is the total volume in the cuvette (0.7 mL), F is the dilution factor, E is the molar extinction coefficient of NADPH at 340 nm (6.22 × 10^−3^ M^−1^ cm^−1^), V_m_ is the sample volume (0.05 mL), and X represents the protein concentration (mg).

#### 2.7.2. Glutathione Peroxidase Enzyme Activity

Glutathione peroxidase activity was measured using spectrophotometry according to the method outlined by Lawrence and Burk (1976) [48], with slight modifications [47]. This method uses cumene hydroperoxide (cumene-OOH, Sigma-Aldrich, Ref. 820502, Madrid, Spain) as the substrate and evaluates the oxidation rate of glutathione (GSH) induced by cumene-OOH in a reaction facilitated by glutathione peroxidase. Briefly, cells were sonicated in 50 mM phosphate buffer at pH 7.4 (previously helium-bubbled), and the samples were then centrifuged at 4 °C. The supernatants were collected, and GPx activity was determined in these samples. The process was monitored by measuring the decrease in absorbance at 340 nm, which corresponded to the oxidation of NADPH (Sigma-Aldrich, Ref. N7505) in the presence of an excess of glutathione reductase (GR). The activity of glutathione peroxidase (GPx) was expressed in mU/mg of protein and calculated using the following equation:

$$GPx\;actitivity\;\left(\frac{mU}{mg\;protein}\right)=GPx\;activity\times \frac{F}{E\times X}$$

where F represents the dilution factor, E is the molar extinction coefficient of NADPH at 340 nm (6.22 × 10^−3^ M^−1^ cm^−1^), and X corresponds to the protein concentration (mg).

#### 2.7.3. Glutathione Content Assay

Glutathione levels were measured using a fluorometric method adapted from Hissin and Hilf (1976) [49], with some modifications [47]. Blood samples were resuspended in 50 mM phosphate buffer containing 0.1 M EDTA (pH 8) and then sonicated.

For GSSG quantification, 10 µL of the sample or standard curve solution and 12 µL of N-ethylmaleimide (NEM, 0.04 M; Sigma-Aldrich, Madrid, Spain) were added into a 96-well flat-bottomed black plate (Thermo Scientific, Madrid, Spain) to prevent the conversion of oxidized glutathione (GSSG) into reduced glutathione (GSH). The plate was incubated for 30 min at room temperature in the dark. After incubation, 178 µL of 0.1 N NaOH (pH 12.5) was added to each well to alkalize the medium. Then, 20 µL of O-phthaldialdehyde (OPT, 1 mg/mL in methanol) was introduced, followed by a 15-min incubation at room temperature in the dark.

For GSH quantification, 10 µL of the sample or standard curve solution was added to the corresponding wells of a 96-well flat-bottomed black plate (Thermo Scientific, Madrid, Spain). Next, 190 µL of 0.1 M phosphate buffer containing 0.05 M EDTA (pH 8) and 20 µL of OPT (1 mg/mL in methanol) were added. The plate was incubated again for 15 min at room temperature in the dark.

Fluorescence was measured at an excitation wavelength of 350 nm and an emission wavelength of 420 nm. The results were expressed as nmol of GSSG and GSH per mg of protein, and the GSSG/GSH ratio was calculated for each sample.

#### 2.7.4. Lipid Peroxidation (TBARS)

Lipid peroxidation was assessed using a commercial kit (BioVision, Mountain View, CA, USA) that detected the formation of malondialdehyde (MDA)-thiobarbituric acid (TBA) adduct [50]. This reaction is based on the interaction between MDA and TBA. Briefly, samples were resuspended in lysis buffer containing 0.1 mM butylated hydroxytoluene (BHT) to prevent additional TBARS formation during sample preparation and heating. The samples were then sonicated and centrifuged at 13,000× *g* for 20 min. Next, 200 μL of supernatant from each sample was mixed with 600 μL of TBA and incubated at 95 °C for 60 min. After incubation, the samples were cooled on ice for 10 min, followed by the addition of 300 μL of n-butanol (Sigma-Aldrich, Madrid, Spain) to extract the TBARS molecules into the organic phase. After centrifugation, 200 μL of the upper organic phase was transferred to a 96-well microplate, and the absorbance was measured spectrophotometrically at 532 nm. The results were expressed as nmol of TBARS per mg of protein.

### 2.8. Protein Concentration

Proteins were quantified using a commercial bicinchoninic acid protein assay kit (Sigma-Aldrich, Madrid, Spain). This method relies on the reduction of Cu^2+^ to Cu^1+^ under alkaline conditions. The degree of Cu^2+^ reduction correlated with the protein concentration and was measured spectrophotometrically at 562 nm [47]. The results were reported as mg of protein per mL.

### 2.9. Biological Age

The individual biological age of each volunteer was estimated through a mathematical model, the Immunity Clock, which considers neutrophil chemotaxis and phagocytosis, lymphocyte chemotaxis, natural killer cell activity, as well as lymphoproliferation ability. This model was developed by multiple linear regression (MLR) with the SPSS 21.0 program (IBM SPSS Statistics) using a stepwise method, in which the variable most useful for prediction was selected and the variables most fitting with the observed lifespan, considering those previously introduced, were successively selected with a threshold of *p* < 0.05 [4].

### 2.10. Hormonal Assays: Cortisol and DHEA

Plasma cortisol was measured using a Cortisol ELISA kit (ADI-900-071, Enzo Life Sciences, Exeter, UK), and plasma dehydroepiandrosterone (DHEA) was determined using a DHEA ELISA kit (ADI-900-093, Enzo Life Sciences, UK). The results were expressed in µg cortisol/mL of plasma and µg DHEA/mL of plasma.

### 2.11. Safety

All participants were monitored during the experiment, and adverse events were recorded.

### 2.12. Statistical Analysis

The data are represented as the arithmetic mean and standard deviation. The statistical analysis was carried out using the SPSS 21.0 program. All tests were two-tailed, with a significance level of α = 0.05. First, it was checked whether the data from each test followed a normal distribution using the Kolmogorov–Smirnov test, and the homogeneity of variances was verified using the Levene test. Subsequently, a comparison of means was made using a dependent samples t-test. It was considered that there were no significant differences in cases where the *p*-value was greater than 0.05. Figures were built using GraphPad Prism 8 Software (LLC, San Diego, CA, USA).

## 3. Results

**Figure 1:**
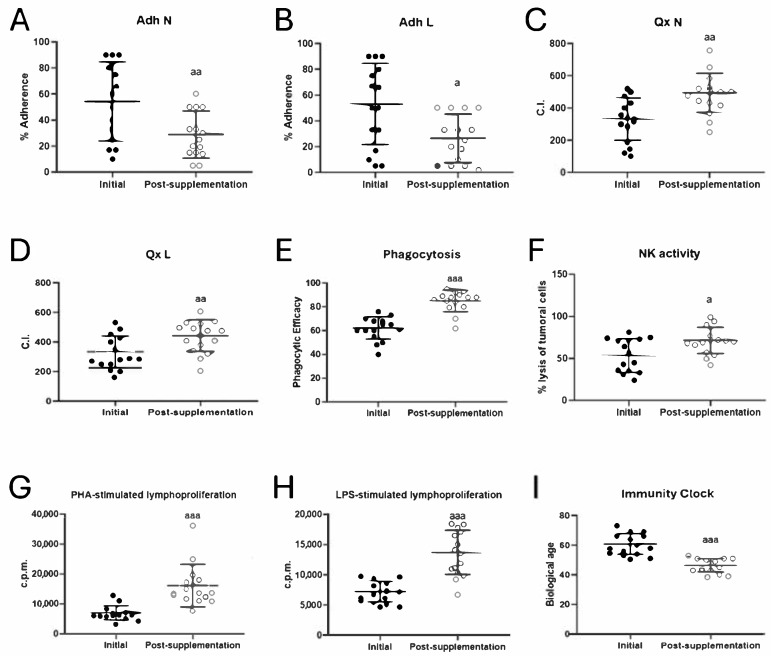
Changes in immune function parameters and biological age before (initial) and after taking UNAMINA (post-supplementation) daily for 1 month (N = 15). (**A**) Neutrophil adherence (%); (**B**) lymphocyte adherence (%); (**C**) neutrophil chemotaxis (CI); (**D**) lymphocyte chemotaxis (CI); (**E**) phagocytic efficacy (% of neutrophils that have phagocytosis of at least one latex particle); (**F**) natural killer cell cytotoxic activity (NK, % tumor cell lysis); (**G**) proliferative capacity of lymphocytes in response to PHA in accounts per minute (c.p.m); (**H**) proliferative capacity of lymphocytes in response to LPS in accounts per minute (c.p.m); (**I**) biological age. a: *p* < 0.05, aa: *p* < 0.01, aaa: *p* < 0.001, respect to its initial value. CI: chemotaxis index; PHA: phytohemagglutinin; LPS: lipopolysaccharide.

To evaluate the effects of taking the oral supplement UNAMINA daily on the immune system, several functions of peripheral neutrophils and lymphocytes were measured. The results obtained for these functions of neutrophils (adherence, chemotaxis, and phagocytic capacities) and mononuclear cells, principally lymphocytes (adherence, chemotaxis, antitumor cytotoxic activity of NK cells, and lymphoproliferative response to the mitogens PHA and LPS), are shown in Figure 1.

The adherence capacities of neutrophils and lymphocytes (Figure 1A,B) showed a decrease in their values (*p* < 0.01 and *p* < 0.05) after taking UNAMINA for 1 month. However, neutrophil and lymphocyte chemotaxis (Figure 1C,D) showed an increase in their values (*p* < 0.01), as well as phagocytic efficacy (*p* < 0.001; Figure 1E) and index (Initial: 206 ± 73; post-supplementation: 376 ± 179; *p* = 0.000), NK tumoral lysis (*p* < 0.05; Figure 1F), PHA- (Figure 1G) and LPS-stimulated (Figure 1H) lymphoproliferation (*p* < 0.001) after taking the oral supplement studied daily for 1 month.

The results of the rate of aging, or biological age, calculated employing the Immunity Clock developed using the values obtained in five immune functions (neutrophil chemotaxis and phagocytosis, lymphocyte chemotaxis, natural killer cell activity, and lymphoproliferation ability [4]), are shown in Figure 1I. The biological age, after taking UNAMINA daily for 1 month, decreased significantly (*p* < 0.001), with a decrease of 15 ± 7 years.

**Figure 2:**
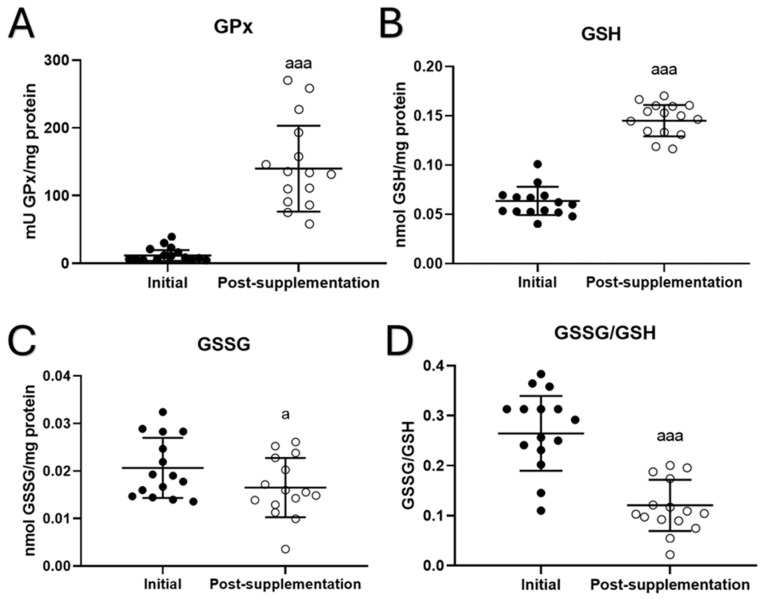
Changes in oxidative parameters before (initial) and after taking UNAMINA (post-supplementation) daily for 1 month in total blood cells (N = 15). (**A**) Enzymatic activity of glutathione peroxidase (mU GPx/mg protein); (**B**) concentration of reduced glutathione (GSH) in nmol/mg protein; (**C**) concentration of oxidized glutathione (GSSG) in nmol/mg protein; (**D**) GSSG/GSH ratio. a: *p* < 0.05, aaa: *p* < 0.001, respect to its initial value.

In total blood cells, several parameters of oxidative stress were analyzed. The results for antioxidant defenses as well as oxidants and oxidative damage to lipids are shown in Figure 2. Increases in the activity of the antioxidant enzyme glutathione peroxidase (GPx) (*p* < 0.001, Figure 2A) and the concentration of reduced glutathione (GSH) (*p* < 0.001; Figure 2B) were observed after taking the UNAMINA supplement for 1 month. A decrease in the oxidant evaluated (GSSG) (*p* < 0.05; Figure 2C) was also found after taking the UNAMINA supplement. The GSSG/GSH ratio, a great marker of oxidative stress, also decreased (*p* < 0.001; Figure 2D). No statistically significant differences were observed in the activity of glutathione reductase (initial = 48 ± 21 mU/mg protein, post-supplementation = 35 ± 19 mU/mg protein) and the concentration of TBARs (initial = 15 ± 4 nmol/mg protein, post-supplementation = 17 ± 3 nmol/mg protein).

**Figure 3:**
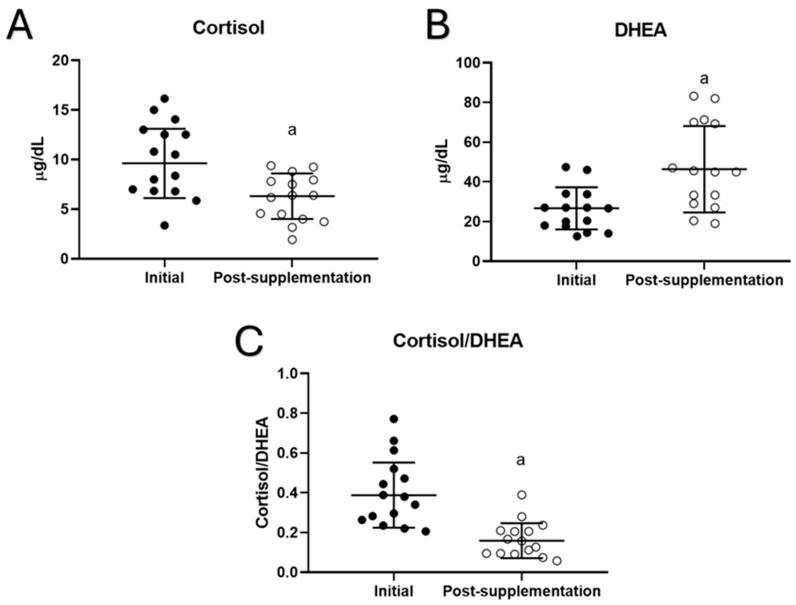
Changes in plasma concentrations of (**A**) cortisol (µg/dL) and (**B**) dehydroepiandrosterone (DHEA, µg/dL), as well as the values of (**C**) the cortisol/DHEA ratio in the participants before (initial) and after taking UNAMINA (post supplementation) for 1 month (N= 15). a: *p* < 0.05, concerning its initial value.

Concerning the effect of taking UNAMINA supplementation daily for 1 month on stress hormones such as cortisol and DHEA, their plasma concentrations were quantified, and the cortisol/DHEA ratio was calculated in volunteers. The results (Figure 3) showed that after supplementation with UNAMINA, participants had a significant decrease in plasma cortisol concentration (*p* < 0.05; Figure 3A), as well as a significant increase in plasma DHEA concentration (*p* < 0.05; Figure 3B). In addition, a decrease in the cortisol/DHEA ratio (*p* < 0.05) was observed (Figure 3C).

It is also important to note that none of the participants showed any adverse events during the experiment. And no significant changes were observed in the quality of sleep, diet, or physical activity of the participants during the experiment. Similarly, no statistically significant changes were obtained in the Perceived Stress Scale and Hamilton’s anxiety rating scale after 1 month of UNAMINA supplementation.

## 4. Discussion

This work describes for the first time that a short period (1 month) of supplementation with a combination of 39 bioactive compounds (glycine-rich collagen precursors, antioxidants, vitamins, and others) improves several relevant functions of peripheral blood immune cells, the redox state, and biological age, as well as hormones related to the stress response in postmenopausal women.

It is known that there is an age-related functional deterioration in immune cells such as lymphocytes, phagocytes (neutrophils), and NK cells. Thus, decreased chemotaxis of neutrophils and lymphocytes, phagocytosis of neutrophils, antitumor cytotoxicity of NK cells, and lymphoproliferative response to antigens or mitogens have been observed [3]. On the contrary, the adherence of lymphocytes and phagocytes to the endothelium, which is the first event that occurs in the immune and inflammatory response, is a function that increases with aging because of the stimulation of adhesion molecule expression produced by the higher presence of reactive oxygen species [44,51,52]. Similarly, previous studies have shown that these immune functions suffer accelerated deterioration during menopause in both humans [38,39,53,54] and mouse models [37,55,56]. However, the results of this study show that, after taking the UNAMINA supplement daily for 1 month, the functions of lymphocytes and neutrophils improved. These results agreed with those obtained using different components of UNAMINA. Thus, glycine-supplemented diets (a constituent of UNAMINA) increased the immune response, had antioxidative and anti-inflammatory effects, and improved psychiatric symptoms in humans and animals [57,58,59]. Also, dietary supplementation with essential amino acids, relevant constituents of UNAMINA, promoted an enhanced immune response and controlled the oxidative state [60,61]. Supplemented diets with adequate levels of antioxidants (vitamin C and vitamin E) can slow down or prevent the general physiological deterioration associated with age progression and, in particular, immunosenescence [2,7,17]. Moreover, it has been seen that supplementation with vitamin C, one of the major constituents of UNAMINA C, normalized monocyte adhesion [62] and improved immune functions [16].

The improvement in immune functions observed after 1 month of supplementation explains the rejuvenation of the biological age of these women since five of the functions mentioned (neutrophil chemotaxis and phagocytosis abilities, lymphocyte chemotaxis and proliferation capacities, as well as NK cell cytotoxic activity) are included in the mathematical model, the Immunity Clock, with which the biological age was calculated [4]. This slowdown in the rate of aging was observed in all of the participants, with an average of 15 ± 7 years. A lower biological age, which indicates a slower aging process, is associated with increased life expectancy. In fact, maintaining a younger biological age may help individuals to lower their risk of age-related diseases and increase their overall lifespan [2,3,4,6]. Previous studies have shown that certain nutritional supplements, some of which are constituents of UNAMINA (including glycine, tryptophan, vitamins, or antioxidants), can extend the life expectancy of healthy individuals [59,63,64,65].

During aging, the production of oxidant compounds is higher than the antioxidant defense capability, thus establishing oxidative stress, which is the basis of aging [5] and immunosenescence [2] as well as accelerated aging in menopausal females [40,56,66,67]. We have observed in previous results that antioxidant defenses in blood cells, such as GPx activity and GSH concentrations, were lower in men and women aged 50–65 years than in those in cells of persons aged 30–49 years, whereas the GSSG amounts were higher [6]. However, postmenopausal women who took UNAMINA daily for 1 month improved their antioxidant defenses (GPx activity and GSH concentration), and oxidant compounds amounts decreased (GSSG concentration). Multiple compounds contained in the oral supplement UNAMINA, such as glycine, vitamin C, and some essential amino acids (methionine and tryptophan), have antioxidant effects, which would explain the results obtained [15,16,61,64].

Inadequate stress response is accompanied by a deterioration of homeostatic systems and further aging [68]. Although stressors have very heterogeneous effects on individuals, it seems clear that chronic stress is associated with immunosenescence [20]. Among the most relevant stress hormones are cortisol and dehydroepiandrosterone (DHEA). Cortisol and DHEA have opposite effects on the immune system; while cortisol causes immunosuppression and its concentration increases with age, DHEA antagonizes the effects of cortisol, with an immunomodulatory effect, and its concentration decreases with age [69]. In addition, this decrease is associated with increased oxidative stress [69]. In this context, the neuroendocrine hypothesis of immunosenescence considers that, because of aging and chronic stress, an imbalance is established in the cortisol/DHEA ratio, which is the main determinant of the immune changes observed during advanced age [69]. Moreover, hormone profile changes during menopause include a decrease in the production of DHEA [70] and an increase in the production of cortisol [71]. However, the results of this study showed that postmenopausal women who took the dietary UNAMINA supplement for 1 month experienced a decrease in basal cortisol concentration and an increase in the concentration of DHEA in plasma. In this way, daily intake for 1 month of UNAMINA maintained an adequate cortisol/DHEA ratio, explaining the restorative effect on immunosenescence and redox state by the dietary supplement studied. These results are supported by previous studies showing that some antioxidant compounds, such as B vitamins [30,31,32] or N-acetyl cysteine [72], one of the constituents of the UNAMINA supplement, can affect the HPA axis and consequently decrease the high basal production of cortisol.

Despite the promising findings, this study presents several limitations that must be considered when interpreting the results. Firstly, the sample size is small, which limits the generalizability of the findings to a broader population. Additionally, the lack of a placebo control group prevents ruling out the influence of other uncontrolled factors on the observed effects. Also, a key limitation of the study is the absence of traditional clinical markers such as hematocrit, lipid profiles, and metabolic parameters, as well as the inability to determine the individual impact of UNAMINA’s components or whether the detected benefits are the result of a synergistic effect. Moreover, the study duration was limited to one month, so it cannot be concluded whether the effects persist in the long term. Finally, since the research focused exclusively on postmenopausal women, it is not possible to extrapolate these results to other population groups, such as men or women in different life stages. To overcome these limitations, future studies should include placebo-controlled clinical trials, a larger population sample, extended follow-up, and mechanistic analyses that can elucidate the specific effects of each component on immunosenescence and aging.

With these limitations in mind, the present study provides evidence that taking the dietary UNAMINA supplement daily for only 1 month is a good strategy to effectively slow down the aging process, neutralizing the accelerated aging of the menopausal period, and thus achieving healthy longevity.

## 5. Conclusions

In conclusion, one-month supplementation with UNAMINA, a combination of 39 bioactive compounds, was associated with significant improvements in immune function, redox balance, stress-related hormones, and biological age in postmenopausal women. Specifically, supplementation enhanced lymphocyte and neutrophil functions and thus decreased biological age, as estimated by the “Immunity Clock”. Additionally, it increased antioxidant defenses and decreased levels of oxidant compounds such as GSSG. Moreover, it modulated the hormonal response to stress, reducing cortisol concentrations and increasing those of DHEA. These positive effects on health markers suggest that UNAMINA may be a promising strategy for promoting healthier aging in postmenopausal women.

However, the lack of a parallel control group in the pre-post intervention design limits the ability to attribute these effects solely to the supplement. Future studies with control groups, larger sample sizes, and longer follow-up periods are needed to confirm and further validate these results.

## Figures and Tables

**Table 1:** Demographic characteristics and lifestyle of participants.

Characteristic	Pre (N = 15)	Post (N = 15)
Female, sex, n (%)	15 (100)	
Age, mean (SD), years	59.3 (6.5)	
Age group, n (%) 40–50 years 51–60 years 61–70 years 71–80 years	1 (6.7) 10 (66.7) 3 (20) 1 (6.7)	
Employment status, n (%) Full-time work Part-time work Unemployed Retired Not working for other reasons	13 (86.7) 0 (0) 0 (0) 2 (13.3) 0 (0)	
Smoking regularly ^1^, n (number of cigarretes/day) Alcohol intake, regularly ^2^, n (%)	1 (15) 0 (0)	
Sleep quality Poor (1–4), n (%) Acceptable (5–7), n (%) Optimal (8–10), n (%)	1 (6.7) 13 (86.7) 1 (6.7)	0 (0) 13 (86.7) 2 (13.3)
Balanced diet Poor (1–4), n (%) Acceptable (5–7), n (%) Optimal (8–10), n (%)	1 (6.7) 12 (80) 2 (13.3)	1 (6.7) 12 (80) 2 (13.3)
Physical activity Poor (1–4), n (%) Acceptable (5–7), n (%) Optimal (8–10), n (%)	2 (13.3) 12 (80) 1 (6.7)	1 (6.7) 13 (86.7) 1 (6.7)
Perceived stress, mean score obtained in the survey (SD) Score group, n (%) Low stress (0–13) Moderate stress (14–26) High stress (27–40)	19.25 (4) 11 (73.3) 4 (26.7) 0 (0)	18.75 (4.5) 11 (73.3) 4 (26.7) 0 (0)
Hamilton Anxiety Scale, Mean Score (SD) Score group, n (%) Mild anxiety or no anxiety (0–17) Moderate anxiety (18–24) Severe anxiety (25–30) Very severe anxiety (31–56)	13.43 (9.3) 13 (86.7) 2 (13.3) 0 (0) 0 (0)	10.92 (8.7) 13 (86.7) 2 (13.3) 0 (0) 0 (0)

^1^ Smoking regularly was defined as smoking almost every day during the last year. ^2^ Intake of alcohol, regularly, was defined as drinking at least 1 glass of alcohol every day.

**Table 2:** Medical history of participants.

Characteristic	Overall (N = 15)
Family history, n (%) None Aneurysm Cancer (larynx, lung, lymphoma) Myocardial infarction	7 (46.7) 2 (13.3) 4 (26.6) 2 (13.3)
Previous illnesses (more than 5 years ago), n (%) None Ovarian Cancer Endometriosis Arrhythmia	12 (80) 1 (6.7) 1 (6.7) 1 (6.7)
Operation (more than 5 years ago), n (%) None Total Hysterectomy Cholecystectomy Appendicitis Herniated discs Cesarean section Osteochondroma	8 (53.3) 2 (13.3) 1 (6.7) 1 (6.7) 1 (6.7) 1 (6.7) 1 (6.7)
Blood pressure, n (%) Low Normal High	3 (20) 12 (80) 0 (0)
Daily medication, n (%) Nothing Hormone Replacement Therapy Probiotics supplement Antiarrhythmic medication	10 (66.7) 2 (13.3) 1 (6.7) 1 (6.7)
Frequent fainting/seizure, n (%) Genitourinary tract diseases, n (%) Skin diseases, n (%) Respiratory diseases, n (%)	0 (0) 0 (0) 0 (0) 0 (0)
Gastrointestinal diseases and discomfort, n (%) None Celiac disease/gluten intolerance Gastroesophageal Reflux	12 (80) 2 (13.3) 1 (6.7)
Allergy (food, drugs), n (%)	3 (20)
Autoimmune/endocrine diseases, n (%) None Hypothyroidism	13 (86.7) 2 (13.3)
Oral health Good (daily care/no missing parts), n (%) Bad n, (missing parts) (%)	14 (93.3) 1 (3 pieces) (6.7)

**Table 3:** The ingredient list and the quantities contained in UNAMINA A, B, and C.

Ingredients	Weight (g)
**UNAMINA A (g)**	
Glycine	5.00 g
L-lysine	0.99 g
L-arginine	0.50 g
L-proline	0.50 g
L-leucine	0.25 g
L-isoleucine	0.25 g
L-valine	0.25 g
L-tryptophan	0.05 g
Hydrolyzed collagen	0.50 g
Magnesium citrate	0.35 g (15% NRV)
Calcium carbonate	0.30 g (15% NRV)
Methyl-sulfonyl-methane	0.50 g
**UNAMINA B [Weight per dose 1 capsule (mg) amount supplied NRV]**	
Magnesium oxide	93.28 mg
L-ornithine HCl	50 50 -
L-beta-alanine	50 50 -
N-acetyl L-cysteine	50 50 -
L-threonine	50 50 -
L-histidine	50 50 -
L-glutamine	50 50 -
L-taurine	50 50 -
Ferrous fumarate	42.59 14 100%
Chondroitin sulphate	40 40 -
Glucosamine sulphate	40 40 -
Zinc sulphate hydrate	27.45 10 100%
Niacin (B3)	16 16 100%
Magnesium stearate	10.123 - -
Pantothenic acid (B5)	6.5221 6 100%
Manganese sulphate	6.152 2 100%
Sodium hyaluronate	5 5 -
Pyridoxine (B6)	1.694 1.4 100%
Riboflavin (B2)	1.4 1.4 100%
Thiamine (B1)	1.39854 1.1 100%
Folate (B9)	0.2 200 µg 100%
Sodium selenite	0.1319 55 µg 100%
Biotin (B8)	0.05 50 µg 100%
Cobalamin (B12)	0.0025 2.5 µg 100%
**UNAMINA C [amount (g) provided per daily dose (2 sachets)]**	
Citric acid	1.5 g
Malic acid	1.5 g
Ascorbic acid	1.0 g
	1000 mg vitamin C (1250% NRV)

## Data Availability

All data described are in the manuscript.

## Abbreviations

The following abbreviations are used in this manuscript:

c.p.m.	counts per minute
CI	chemotaxis index
DHEA	dehydroepiandrosterone
EDTA	ethylenediaminetetraacetic acid
fMLP	N-formyl methionyl-leucyl-phenylalanine
GPx	glutathione peroxidase (enzyme)
GR	glutathione reductase (enzyme)
GSH	reduced glutathione
GSSH	oxidized glutathione
HPA	hypothalamus-pituitary-adrenal axis
LDH	lactate dehydrogenase (enzyme)
LP	lymphoproliferation
LPS	lipopolysaccharide
MLR	multiple linear regression
NK	natural killer
OPT	O-phthaldialdehyde
PHA	phytohemagglutinin
PBS	phosphate-buffered saline
PE	phagocytic efficacy
RPMI	Roswell Park Memorial Institute culture medium
SD	standard deviation
TBA	thiobarbituric acid
TBARS	thiobarbituric acid reactive substances
NRV	nutrient reference values
